# RNA editing in mesothelioma: a look forward

**DOI:** 10.1098/rsob.200112

**Published:** 2020-10-14

**Authors:** Ananya Hariharan, Suna Sun, Martin Wipplinger, Emanuela Felley-Bosco

**Affiliations:** Laboratory of Molecular Oncology, Department of Thoracic Surgery, Lungen- und Thoraxonkologie Zentrum, University Hospital Zurich, Sternwartstrasse 14, 8091 Zurich, Switzerland

**Keywords:** mesothelioma, RNA editing, adenosine deaminase acting on double-stranded RNA, type 1 interferon signalling

## Abstract

RNA editing is a post-transcriptional process increasing transcript diversity, thereby regulating different biological processes. We recently observed that mutations resulting from RNA editing due to hydrolytic deamination of adenosine increase during the development of mesothelioma, a rare cancer linked to chronic exposure to asbestos. This review gathers information from the published literature and public data mining to explore several aspects of RNA editing and their possible implications for cancer growth and therapy. We address possible links between RNA editing and particular types of mesothelioma genetic and epigenetic alterations and discuss the relevance of an edited substrate in the context of current chemotherapy or immunotherapy.

## 1. Introduction

Malignant mesothelioma (reviewed in [1,2]) is a rapidly fatal and highly resilient tumour arising in the thin layer of tissue known as the mesothelium, which has mesodermal origins and covers many of the important internal organs like the lungs (pleural mesothelioma), peritoneal cavities (peritoneal mesothelioma), the sacs surrounding the heart (pericardial mesothelioma) and the testis (tunica vaginalis mesothelioma). Although mesothelioma is a rare cancer, its incidence is still rising; hence, research aimed at better understanding of the biology of the disease is still necessary. Since the seminal experiments of Wagner [3], exposure to asbestos has been clearly identified as the cause of mesothelioma. We recently observed in an experimental animal model of asbestos-induced mesothelioma development [4], that asbestos increased the levels of RNA mutations and the most abundant changes were A to G mutations, probably resulting from the hydrolytic deamination of adenosine downstream of adenosine deaminase editing activity [5] (I is detected as G in RNA sequencing).

While several recent reviews are available on RNA editing [6–12], in this review we shall focus on possible implications of RNA editing in mesothelioma, a subject that is beginning to be explored.

## 2. RNA editing by adenosine deaminases acting on RNA in mesothelioma

The term ‘RNA editing’ refers to enzymatic post-transcriptional events that increase transcript diversity by altering nucleotide sequences through insertion, deletion or conversion of a nucleotide. The most frequent event is the hydrolytic deamination of adenosine [13]. This activity was discovered by serendipity, when investigators observed failure of using antisense RNA technology to study embryonic development, because of instability of RNA duplexes. Loss of RNA's base-pairing properties, hence loss of double-strand RNA (dsRNA) structure was due to the conversion of adenosines (A) to inosines (I) [14,15]. This activity allowed the identification of the adenosine deaminases acting on RNA (ADAR) family [16,17].

**Figure 1.**
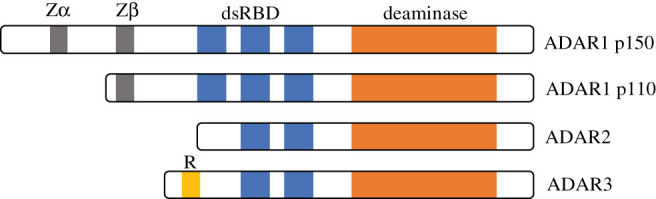
ADARs share common functional domains—the dsRBDs and the catalytic deaminase domain. ADAR1 contains three copies of the dsRBD, while ADAR2 and ADAR3 possess two copies each. ADAR1 has two isoforms, p150 and p110, which are produced by alternative splicing and the usage of different promoters. ADAR1 has Z-DNA-binding domains at the N-terminal. Zβ is common to both ADAR1 p150 and ADAR1 p110, but Zα is unique to ADAR1 p150. ADAR3 contains an arginine-rich single-stranded RNA-binding domain (R) at the N-terminus.

Vertebrates have two catalytically active (ADAR1 and ADAR2, previously called ADAR and ADARB1, respectively) and one catalytically inactive (ADAR3) ADAR proteins, while *Drosophila* (for example) has only one (ADAR2 orthologue). All ADAR proteins contain dsRNA-binding domains (dsRDBs), which allow sequence specificity [18] and a C-terminal deaminase domain (figure 1). As will be discussed later, ADAR1 has additional domains (z-DNA binding).

RNA editing by ADAR occurs mostly in non-coding regions where inverted repeat sequences are likely to form dsRNA structures, which function as the substrate. In mouse normal monocytes or tissues, 32–73% of all editing events occurs in short-interspersed elements (SINEs) and 9–27% in long terminal repeat (LTR) retrotransposons [19,20], while in human normal monocytes or tissues, 43–96% of all editing events occurs in SINE and 7.4–2.4% in LTR retrotransposons [19,21], depending on cells and tissues analysed. In mouse and human monocytes, 44% and 37%, respectively, editing occurs in non-repetitive elements.

SINEs are recurrent elements, which propagate through the reverse transcription of an RNA intermediate. Human SINE, Alu, is a 300 nt-long retroelement constituting 10% of the human genome. LTR retrotransposons include endogenous retroviruses (ERVs) and their integration is mediated by an integrase [22]. Altogether SINE and LTR retrotransposon regions cover 42% and 37% of the genome in human and mice, respectively [23,24]. Taking into account that 70% of the genome is transcribed and only 2% of the genome encodes for proteins [23,25–28], this may explain the reason why the vast majority of editing sites in human and primates are in inverted repeat SINE (Alu elements in human) and ERV which forms stable dsRNA structures and are largely in non-coding regions of the genome [29,30]. Using The Cancer Editome Atlas (TCEA; http://tcea.tmu.edu.tw), a resource characterizing editing events across 33 cancer types in The Cancer Genome Atlas (TCGA) [31], we determined that 394 editing events occur in Alu elements located in 3′-UTR regions in more than 80 mesothelioma samples, indicating potential gene expression regulation by altering, for example, miR-targeting or interaction with RNA-binding proteins (discussed in a later section).

The formation of a homodimer is necessary for RNA editing activity [32–34]. Heterodimer formation between ADAR1 and ADAR2 remains controversial; however, when present it leads to reduction in the specificity of the enzyme for some RNA editing sites [32,35]. Dimer formation is mediated by the double-stranded RNA-binding domain (dsRBD) [36].

ADAR3 expression has not been detected in either the experimental animal model of mesothelioma development or in human mesothelioma; therefore, it will not be further discussed in this review.

Although ADAR1 and ADAR2 are mostly reported as ubiquitous [37], a recent single-cell transcriptome analysis of 20 mouse organs [38] (https://tabula-muris.ds.czbiohub.org/) indicates that not all cells express these genes. In the study, single cells from a given organ were identified and sorted using cell surface markers. In the diaphragm (which is often used in studies on mesothelium because this organ is covered by mesothelial tissue on the peritoneal surface), *Adar2* is expressed mostly in some mesenchymal stem cells (Sca-1^+^, CD31^−^ and CD45^−^), while similar levels of *Adar1* are expressed in some endothelial cells (CD31^+^ and CD45^−^). Importantly, in the context of this review, we have described expression of Sca-1 (also called Ly6A), a gene induced by type 1 interferon (IFN) [39], in putative mesothelioma stem cells, which are enriched upon therapy in an experimental mouse model [40]. In addition, in the experimental animal model of asbestos-induced mesothelioma development mentioned above [4], we observed a significant 3.9-fold increase of *Adar1* expression in inflamed tissue compared with sham and more than twofold increase in tumours compared with inflamed tissues. Intriguingly, *Adar2* showed a significant, more than twofold increase in tumours compared with inflamed tissues, but its expression was not significantly changed between sham and inflamed tissues.

Analysis of TCGA mesothelioma data revealed [4] that high expression of *ADAR2* is associated with worst overall survival, supporting the idea that RNA editing is relevant in mesothelioma as it is in other cancers (reviewed in [8,9,41,42]). In addition, decreased expression of *ADAR2* has been observed upon Yes-associated protein (YAP) silencing in mesothelioma cells, which resulted in decreased cell growth [43], providing a possible mechanism behind the TCGA data associating high *ADAR2* expression with worst overall survival. YAP is a transcriptional co-activator after interaction in the nucleus with the TEAD family of transcription factors [44], resulting in the induction of the genes promoting cell proliferation and inhibition of apoptosis [44–47]. The Hippo cascade regulates YAP via *large tumour suppressor homologue 1/2* (LATS1/2)-dependent phosphorylation and subsequent cytosolic sequestration [46,48,49]. In mesothelioma, due to the *neurofibromatosis 2* or *LATS2* loss, Hippo signalling becomes dysregulated [50–52]. Furthermore, in a recent study, silencing *ADAR2* in one mesothelioma cell line resulted in reduced cell proliferation, invasiveness and motility, while overexpression of ADAR2 with a mutated dsRBD showed the opposite effect [53], consistent with a dominant negative effect of mutant overexpression.

## 3. ADAR1: type 1 IFN-dependent and -independent effects and implications for mesothelioma therapy

There are two isoforms of ADAR1: a constitutively expressed nuclear p110 and a longer, cytosolic and nuclear, IFN-inducible p150 protein, containing two complete z-DNA-binding domains, while only one is found in p110 [54].

**Figure 2.**
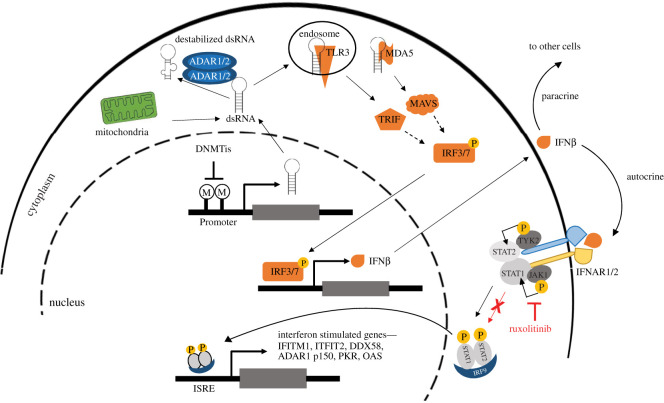
dsRNA-driven activation of IFN signalling. Increased levels of dsRNA formation triggered by DNA methyltransferase inhibitors (DNMTis) or derived from mitochondria cause a type I IFN response via dsRNA sensors such as MDA5 and TLR3. Sensors are recognized by TRIF/MAVS leading to phosphorylation and activation of IRF3/7 which translocates to the nucleus and drives the expression of type I IFN (represented here by IFN-β). IFN-β is secreted and acts in an autocrine or paracrine manner. Upon binding to the IFNAR1/2 receptor, transactivation of JAK1 and TYK2 leads to the phosphorylation of STATs and the formation of IRF9/p-STAT1/p-STAT2 complex which acts as a transcription factor driving the expression of ISGs through the binding of IFN-sensitive response elements (ISREs).

The essential role of Adar1 has been established using several genetically modified mouse models. *Adar1*-deficient mice, lacking either exons 12–13, exons 7–9 or exons 2–13, die at embryonic day 11.5–12 [55,56] due to decreased hepatoblast number, alteration of fetal liver structure and defects in haematopoiesis. A later study has demonstrated an increase of type 1 IFN and IFN-stimulated genes (ISGs) in haematopoietic stem cells and erythroid cells [57]. In *in vivo* mouse models, the function of *Adar1* p150-mediated editing is to prevent endogenous dsRNA-dependent activation of innate immune receptors such as mitochondrial antiviral signalling adaptor protein (MAVS) [58] or *IFIH1*-encoded melanoma differentiation-associated protein 5 (MDA5) [59,60] (figure 2). Indeed, embryonic lethality is rescued in double ADAR1/dsRNA sensor mutants, although mice survive only until birth. However, mice lacking exons 7–9 rescued by *Mavs*^−/−^ and *Ifih*^−/−^ have an intermediate phenotype and live longer compared with *Adar1*-deficient mice lacking exons 2–13; *Mavs*^−/−^ [61]. Since the deletion in exons 7–9 covers the third dsRDB and part of the deaminase domain, these observations indicate an editing-independent function of Adar1.

dsRNAs, like other nucleic acids, are part of the signals recognized by pattern recognition receptor family which are able to activate innate immunity via the production of type 1 IFN [62]. This is consistent with the aberrant activation of innate immune receptors observed in a spectrum of immune disorders, such as systemic lupus erythematosus or Aicardi–Goutières syndrome, a disease characterized by severe changes in the brain and neurological function, and it has been linked to mutations inducing loss of ADAR function [63] or gain of function in MDA5 [64].

Mesothelioma is the sixth of 31 cancer types with most prevalent ISG 38 gene signature [65]. Importantly, in the context of mesothelioma, type 1 IFN signature is linked to both clinical outcome and specific driver mutations. A recent large-scale study has comprehensively characterized most genetic alterations and four distinct molecular profiles in malignant pleural mesothelioma (MPM), which have been called epithelioid (which actually include mostly only pure epithelioid histotype), biphasic-epithelioid, biphasic-sarcomatoid and sarcomatoid [66]. It extends the histopathological classification separating epithelioid, sarcomatoid and biphasic of mesothelioma (reviewed in [2]). Based on the mRNA expression profile, tumours are clustered into four groups [67] in a parallel study performed by TCGA consortium [68]. Pathway enriched analysis of genes expressed in the clusters revealed among others, enrichment of reactome antiviral mechanism by ISG in one of the clusters, and this is confirmed in the epithelioid group of Bueno *et al.* [66]. Patients with this profile have a better clinical outcome [69]. The integrative multiomics analysis of mesothelioma TCGA [68] data revealed that the activation of type 1 IFN has been linked to the status of BRCA-associated protein 1 (BAP1), a gene which is frequently mutated in mesothelioma [2]. In addition, a recent analysis of TCGA public available data revealed a negative correlation between BAP1 expression and a constitutively activated IFN type 1 response [70]. However, the underlying mechanisms are not clear yet, and we shall propose in this review different scenarios. Importantly, a recent study has shown that primary mesothelioma cells maintain the activation of the type I IFN signalling pathway [71].

More recently, targeted *Adar1* deletion in neural crest cells also resulted in the death of mice 10 days after birth due to impairment of neural crest cell differentiation to melanocytes [72]. This may explain why mutations in human ADAR1 are associated with dyschromatosis symmetrica hereditaria [73], an autosomal dominant hyperpigmentation of the hands and feet occurring in Chinese and Japanese families. The majority of these disease-associated mutations are single-allele truncations of ADAR1, and the dominant phenotype seems to be due to haploinsufficiency for ADAR1. In addition, targeted *Adar1*–deletion in neural crest cells is accompanied by upregulation of type 1 IFN-regulated genes. Again, this phenotype seems to be mediated by Mda5/Mavs activation [72]. All these observations point to a role in avoiding dsRNA sensing during embryo development.

The relevance of the z-DNA-binding domain of ADAR1 p150 in the dysregulation of ISG has been recently highlighted by analysis of dsRNA-binding impaired natural variants of this domain (Pro193Ala and Asn173Ser) in the few cases of diseases linked to the loss of an allele of ADAR1 p150 but not ADAR1 p110 [74]. In these cases, the presence of a single allele with natural variants impairing the function allowed researchers to understand that the z-DNA-binding domain contributes to dsRNA binding. z-DNA is a left-handed dsDNA occurring when polymerase or helicases underwind DNA, and the formation of a complex between the z-DNA-binding domain with double-stranded nucleic acids is most rapid with DNA/RNA hybrid duplexes (reviewed in [75]). The z-DNA-binding domain is not necessary for editing [76]. It has been suggested that z-DNA in a specific repetitive sequence would allow anchoring of ADAR1 to dsRNA, causing rapid editing after transcription, and a mutation in the z-DNA-binding domain decreases dsRNA binding [58]. ADAR1 binds to specific sequences through its z-DNA-binding domain in Alu elements, thereby decreasing Alu retrotransposition (reviewed in [75]). The human genome harbours active Alu retrotransposons, mostly within the AluY family, which need to interact with a protein called SRP9/14 for retrotransposition [77], and the binding of ADAR1 prevents this interaction. Retrotransposition has been linked to the occurrence of chromotripsis, a phenomenon characterized by multiple focalized double-stranded DNA breaks resulting in complex genomic rearrangements, by the analysis of a familial germline chromotripsis [78]. Although chromotripsis has been observed in some mesothelioma cases [79,80], it is not yet known whether retrotransposition is involved.

Although the phenotype of *Adar1*-deficient mice can also be due to editing-independent functions, the role of the catalytic function of Adar1 in the phenotype of *Adar1*-deficient mice has been confirmed in studies using mice with an editing knock-in mutation (E861A), which results in disruption of the catalytic activity [59]. Interestingly, compared with full disruption of the gene, knock-in mutation mice are fully viable when crossed with *Ifih*^−/−^ mice, which is consistent with editing activity-independent effects of Adar1. This has been recently confirmed by the observation of downregulation of 40S ribosomal protein RPS3a1 accompanied by upregulation of its pseudogene RPS3a3, thereby affecting ribosomal subunit assembly, in *Adar1*-deficient mice lacking exons 2–13; *Mavs*^−/−^ but not in editing deficient E861A; *Ifih*^−/−^ mice [61].

While most investigations draw attention to the role of ADAR1 in preventing the activation of type 1 IFN, the observation of abnormal kidney development in *Adar1*^−/−^; *Mavs*^−/−^ but not in *Adar1 p150*^−/−^*; Mavs*^−/−^ indicates a specific role for Adar1 p110 in renal development [60]. This is important in the context of mesothelioma because, in the experimental animal model of mice exposed to asbestos, we observed reactivation of developmental organ signalling pathways including the kidneys [4].

Interestingly, in the *Adar*1-deficient models described above, even in the absence of Mda5 there is a mild, non-pathogenic induction of few ISGs by a currently unknown mechanism [60,81]. In addition, some deregulated gene expressions related to the control of cell fate specification during development are also independent of dsRNA sensing by Mda5 [60]. Therefore, nuclear RNA editing of genes expressed during embryo development or genes reactivated in cancer has consequences for both innate immunity and specific innate immunity-independent signalling.

**Figure 3.**
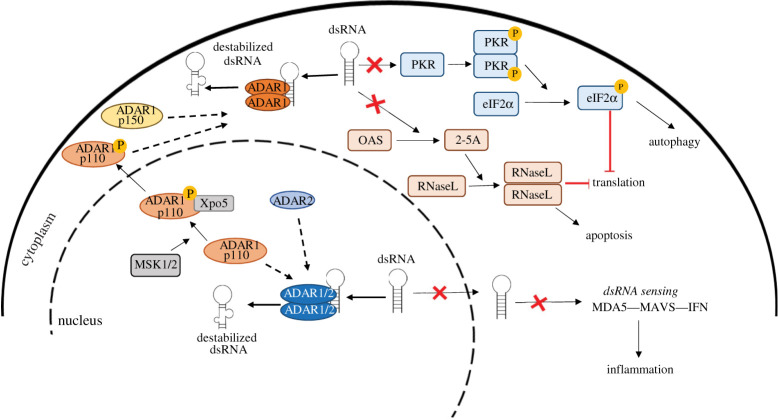
dsRNA is destabilized by ADAR activity. In the nucleus, ADAR1 p110 and ADAR2 are recruited for the destabilization of dsRNA. In the cytoplasm, ADAR1 p150 recognizes dsRNA. In conditions of stress, ADAR1 p110 is phosphorylated by MAP kinase MSK1/2, leading to the recruitment of Xpo5. This allows ADAR1 p110 to be exported into the cytoplasm to work alongside ADAR1 p150. The destabilization of dsRNA by ADAR activity supresses other mechanisms of dsRNA sensing in the cell—(*a*) PKR leading to the activation of eIF2α resulting in the inhibition of translation and autophagy, (*b*) OAS leading to the activation of RNaseL resulting in translational inhibition and apoptosis, and (*c*) MDA5–MAVS leading to IFN production and inflammatory response.

The reason why it is important to maintain the homeostasis in dsRNA sensing is that dsRNA activates the IFN-inducible dsRNA-dependent Ser/Threo protein kinase (PKR) (figure 3), thereby inducing its dimerization, autophosphorylation and the phosphorylation of eIF2α. This has as a consequence induction of autophagy and the inhibition of translation [82,83]. In the absence of ADAR1, cellular dsRNA formed by unedited inverted-Alu repeats was proposed to activate PKR [84]. Breakdown of the nuclear membrane during mitosis and consequent exposure to an excess of nuclear inverted-Alu repeats were also proposed to activate PKR [85]. Additional endogenous ligands include dsRNA from sense–antisense RNA produced by bidirectional transcription in the mitochondria [86], and any damage to the mitochondrial membrane might result in leakage of dsRNA to the cytosol. However, mitochondrial RNA represents less than 15% of dsRNA in HeLa cells [86].

It is important to note that cancer cells express a basal level of PKR making them predisposed to trigger inhibition of translation and downstream events. The destabilizing of dsRNA structures by ADAR activity suppresses dsRNA-activated signalling to avoid growth arrest due to the activity of the other genes and is therefore considered to act as a negative feedback loop [60,84]. dsRNA also increases the activation of IFN-inducible oligoadenylate synthetases (OAS), which produce 2′,5′-oligoadenylate activating RNAse L to degrade rRNA, tRNA and Y-RNA. However, this is observed in some cancer cells only after the addition of type 1 IFN [87].

In the context of mesothelioma, it is important to note that ADAR1 is a target of the type 1 IFN pathway acting as a negative feedback regulator to avoid autoimmunity, an effect which has recently been linked to asbestos amphiboles (reviewed in [88]).

Until now, ADAR1 loss of function in cancer cells has been observed and explored in the context of immunotherapy. Adar1 loss of function has been found as a top candidate to boost immunotherapy in a screen of melanoma cells implanted in mice treated with anti-PD1 [89,90]. Some cancers have spontaneous production of type 1 IFN leading to ISG signature; however, the signal activating the type of IFN response may depend on aberrant DNA species detected by a stimulator of IFN genes (STING) [65]. These cancer cells are sensitive to ADAR1 depletion, and this effect is rescued by depleting PKR [65,91] or overexpressing ADAR1-p150, but not ADAR1-p110. Importantly, total (or in some cases partial) rescue has been observed by overexpression of catalytically inactive E912A ADAR1 p150 [92]. Therefore, in embryogenesis, ADAR1 is essential to avoid signalling downstream of dsRNA, but in cancer cells the primary aim is to avoid growth arrest, and ISG expression seems to be a ‘side effect’ which, nevertheless, can be exploited for immunotherapy [65,90].

More recently, another regulatory role of ADAR1 in the nucleus has been revealed [93]. ADAR1 forms a complex with the Drosha cofactor DGCR8 and competes with its binding to Drosha. The exact role of the ADAR1/DGCR8 complex remains unclear, but it has been suggested that it contributes to the global dysregulation of pri-miRNA processing by Drosha. Monomeric ADARs also complex with Dicer to increase the rate of pre-miRNA cleavage and facilitate miRNA loading onto the RNA-induced silencing complex (RISC) independently of their editing function. Adar1 knockout embryos show a global reduction of mature miRNA abundance and gene silencing, suggesting that the role of ADARs in promoting pre-miRNA processing may dominate [94]. ADAR1 is frequently reduced in metastatic melanoma, which results in dysregulation of more than 100 miRNAs [93]. It is also worth noting that downstream of the activation of YAP, which, as previously mentioned, is frequently activated in mesothelioma (also reviewed in [95]), a decrease in the activity of Drosha has been observed [96]. Therefore, RNA editing and YAP/TAZ activation may converge on profound modification of mature miRNA.

## 4. ADAR2 and its role in genomic stability

ADAR2 is essential for the specific editing of the glutamate receptor 2 subunit, resulting in a change from a genomically encoded glutamine to arginine, thereby varying the permeability of the pore [97]. *Adar2* deficiency results in early lethality due to seizures, and this can be rescued by a point mutation in *Gria2* (*Gria R/R* mice).

ADAR2 is expressed in most mammalian tissue with brain and lung expressing the highest levels [98]. The crossing of *Adar2^−/−^; Gria2 R/R* mice with *Adar1 Δ7–9; Mavs^−/−^* mice showed decreased survival and increased activation of type 1 IFN signalling, indicating a previously unexpected, although small, compensatory contribution of Adar2 in the phenotype of *Adar1 Δ7–9; Mavs^−/−^* [61].

ADAR2 undergoes alternative RNA splicing to yield isoforms that differ in their editing efficiency. Alternative splicing of rat and mouse Adar2 pre-mRNA generates two splice variants that differ by a 10-amino acid splice cassette in their deaminase domains [99]. The human ADAR2 undergoes alternative splicing of the same exon. Alternative splicing in this region of the human transcript inserts a 40-amino acid Alu-J cassette also in the deaminase domain, thereby reducing its catalytic activity by a factor of two [100–102]. In addition, editing of rat Adar2 pre-mRNA regulates its alternative splicing with the generation of 47 nt insert that leads to decreased activity [99].

The physiological significance of alternative splicing of the ADAR2 transcript is not yet known, besides resulting in differential activity. Therefore, it is difficult to interpret the observation that Adar2 expression is increased in developing mesothelioma in asbestos exposed mice and that high ADAR2 expression is associated with worst overall survival in mesothelioma patients [4] until additional functional studies are carried out.

In the context of cancer, where DNA damage repair may be not fully functional, it is worth mentioning that full-length ADAR2 can edit hybrid DNA/RNA duplexes [103]. This activity may have implications for the generation of mutations where DNA/RNA hybrids occur, for example, in R-loops [104].

## 5. Mesothelioma-relevant editing targets

**Figure 4.**
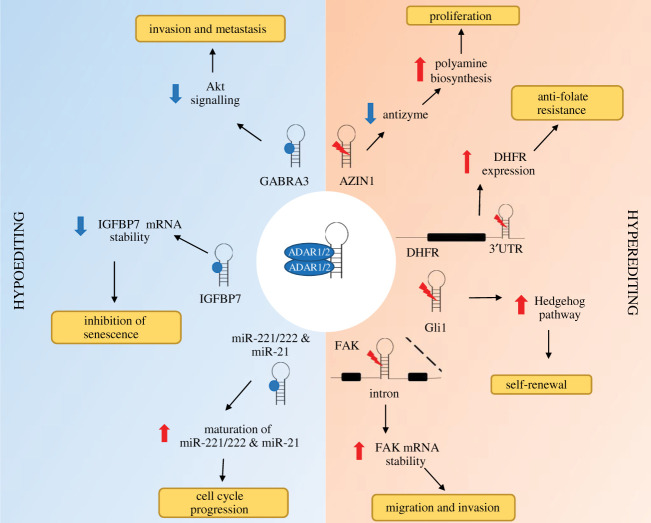
Dysregulation of A-to-I RNA editing promotes cancer progression. ADAR1 upregulation leads to elevated editing within targets, e.g. AZIN1, DHFR, GLI1 and FAK. The increase in A-to-I editing of these targets contributes to cancer development and progression via different cellular pathways. AZIN1 editing increases polyamine biosynthesis resulting in increased cell proliferation. Editing of the 3′-UTR of DHFR increases the stability of the mRNA and hence causes resistance to anti-folates. Edited GLI1 upregulates the Hedgehog pathway and therefore enables cells to self-renew. Edited FAK mRNA degrades slowly causing a build-up of FAK which results in increased cell migration and invasion. Downregulation of ADAR1 editing decreases the editing of GABRA3. Unedited GABRA3 promotes cancer cell invasion and metastasis through upregulation of the Akt pathway. A decrease in ADAR2 editing activity affects targets such as IGFBP7 and miR-221, -222, -21. Unedited IGFBP7 mRNA is highly unstable and results in inhibition of senescence. Hypoediting of miR-221, -222, -21 leads to an increase in their maturation resulting in faster cells cycling. Red mark indicates A-to-I editing, while blue dot indicates an unedited site.

In cancer, both the editing of RNA coding sequences and the destabilizing of dsRNA structures seem important. In most of the cases, it is the increased editing that favours cancer, but there are also examples of decreased editing (figure 4). Editing of *AZIN1*, which encodes an antizyme inhibitor, generates an amino acid change (Ser367Gly) creating an isoform with increased affinity to antizyme, promoting cell proliferation by reducing antizyme-mediated degradation of ornithine decarboxylase and cyclin D1, and has been associated with hepatocellular carcinoma progression [105]. *AZIN1* editing is also observed in mesothelioma, where it can be used as readout of RNA editing activity [4]. Relevant for MPM, where a subset of patients show Hedgehog signalling activation (reviewed in [106]), editing of mRNA encoding for glioma-associated oncogene 1 (GLI-1) results in increased Gli-1 protein stability and maintains tumour initiating cells in leukaemia [107].

In some cases, decreased editing favours some cancers. Editing of insulin-like growth factor-binding protein 7 (IGFBP7), a secreted protein associated with apoptosis and senescence [108], in oesophageal cancer cells, results in an amino acid change protecting the protein from proteolysis [109]. Editing of GABA receptor alpha3 (Gabra3) in breast cancer cells changes an amino acid, thereby reducing its surface expression and decreasing cell migration and invasion, and is accompanied by decreased AKT activation [110]. Importantly, in the context of mesothelioma, *Gabra3* is part of the genes upregulated in total lung tissue after mice exposure to asbestos [111]. Therefore, some editing triggered by asbestos exposure might represent a cell defence reaction against transformation. Finally, ADAR2 editing of pre-miR-221 and 222 results in decreased levels of mature miR-221 and 222 and decreased growth in glioblastoma cells [112]. Importantly in the context of mesothelioma, decreased levels of miR-221 and miR-222 have been documented in mesothelioma [113] and miR-221 high expression is associated with better overall survival in mesothelioma patients [114].

Most editing sites occur in non-coding regions including non-coding regions of transcribed genes. In the context of MPM, which is treated in first-line therapy with cisplatin and antifolates, editing of the 3′-UTR may protect dihydrofolate reductase (DHFR) mRNA from mir-25-3p- and miR-125-3p-induced degradation, leading to resistance to antifolates such as methotrexate and pemetrexed, as it has been recently observed in breast cancer cells [115]. Reduced cell migration has been observed in lung cancer cell lines due to the inhibition of ADAR-mediated RNA editing, and the destabilization of focal adhesion kinase (*FAK*) mRNA was identified as responsible of this phenotype [116]. This might be important in the context of mesothelioma because NF2 alterations, which are frequent in mesothelioma (reviewed in [2]), result in the activation of FAK and mesothelioma cells are sensitive to FAK inhibitors [117–120].

Adenosine-to-inosine conversion can change the sequence of the mature miRNA (including the critical seed sequence) to block target recognition and also change base pairing and hence can reduce the dsRNA structure to interfere with pri-miRNA processing. For example, editing of hairpin structures in pri-miRNA reduces the production of mature miRNA due to the impairment of their processing by Drosha [5,121]. RNA editing of pri-miR-142 by ADAR1 and ADAR2 inhibits its processing by Drosha and also facilitates pri-miR-142 degradation [122].

**Figure 5.**
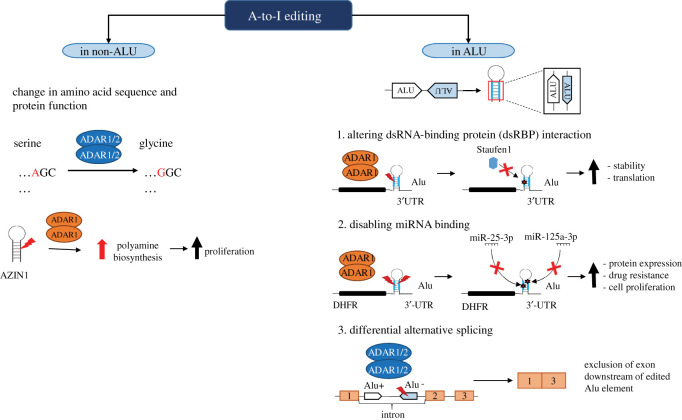
Consequences of A-to-I editing in non-Alu and Alu elements. A-to-I editing leads to an amino acid change from serine to glycine in the coding sequence of AZIN1, resulting in stable activation and consequently an elevated proliferation rate. Alu elements are 200–300 bp-long transposable elements that can be found in two opposite orientations, which are able to form dsRNA structures and serve as targets for ADAR1/2. They can be located in 3′-UTRs as well as in intronic regions. (1) Editing in the 3′-UTR can disable the binding of dsRBP, which can, for example, increase RNA stability and allow translation, as shown in the example for Staufen1. (2) It can also prevent miRNA binding, like for miR-25-3p and miR-125a-3p to DHFR, averting RNA degradation and promoting protein translation. (3) Editing in intronic Alu elements can induce alternative splicing, leading to an exclusion of the exon downstream of the edited Alu elements.

Although the minimal dsRNA length functioning as the substrate for ADAR has been estimated to be 20–22 bp, the longer the dsRNA is, the more editing sites it will acquire [123]. In addition, dsRNA must be at least 30 bp long to elicit an immune response [124]. But, beside ADAR and dsRNA sensors like MDA5, PKR and OAS, there is a plethora of dsRNA-binding proteins (dsRBPs) [125]. Therefore, it is necessary to keep in mind that during its lifetime, a given dsRNA may interact with multiple dsRBPs. The latter are not sequence-specific; therefore, editing is in competition with other dsRBP-induced events (figure 5) [126,127]. In some cases, such as in cells stressed by UV irradiation, ADAR1 p110 is relocalized to the cytosol, where it competes with dsRNA-binding protein Staufen1 to decrease mRNA decay of several genes, including *ATM* and *RAD51*, which are crucial in the DNA damage response [128].

Another example concerns Murine Double Minute 2 (MDM2), an oncogene overexpressed in some mesothelioma tumours, which has been investigated as a therapeutic target in mesothelioma with wild-type p53 [129–131]. MDM2 3′-UTR is edited in all TCGA mesothelioma samples according to the TCEA. ADAR1-dependent editing of MDM2 3′-UTR facilitates nuclear retention of MDM2 mRNA by competing with Staufen1 [132]. The resulting altered translation efficiency likely explains the moderated correlation observed between gene and protein expression in mesothelioma tumours [133].

It has been recently shown that endogenous Alu–Alu inverted repeats (figure 5) deriving from the 3′-untranslated region (UTR) of mRNA trigger inflammation in *Adar1-*deficient state or constitutively active Mda5 [134]. This observation indicates that mere baseline transcription of repetitive elements is able to trigger type 1 IFN response in the absence of efficient RNA editing. Therefore, one could ask whether specific dsRNAs are expressed during embryo development or in cancer.

Intriguingly, a recent study has shown intrinsic, IFN-independent ISG expression in stem cells, and the expressed ISG genes, like members of IFITM family, act on early steps of viral life cycle, while no gene with well-known antiproliferative activity was detected [135]. The molecular origin of the stem-cell-specific and IFN-independent expression of ISG remains elusive beside altered promoter methylation [135]. In this context, it is worth noting that the expression of human ERV HERVK has been observed in embryonic stem cells downstream of OCT4 and SOX2 activity, and is facilitated by demethylating agents, which also increase IFITM1 expression [136]. This observation is especially important because of the current use of viral mimicry in the context of several clinical trials where effects of immune checkpoint inhibitor are tested in combination with demethylating agents, which increase the expression of ERV sequences [137,138]. Interestingly, upon demethylation, dsRNA are also sensed by Toll-receptor-like 3, in addition to previously mentioned dsRNA sensors [137].

From these observations, the question arises whether the activation of type 1 IFN in mesothelioma is due to the reactivation of embryonically active elements, which are able to form dsRNA and which have been silenced.

Within embryonically active elements, which are often hypomethylated (and thereby de-repressed) in cancer, there are ERV sequences, and a recent analysis of TCGA data has revealed upregulation of ERV sequences in several cancer types [139]. DNA methylation at CpG constitutes together with histone methylation the major mechanism of transcriptional control of ERVs (reviewed by Friedli & Trono [140]).

In mammals, CpG methylation is initiated by the de novo methyltransferases including DNMT3a, 3b and is perpetuated across mitosis by the maintenance of DNA methyltransferase DNMT1. DNA demethylation occurs passively during DNA replication [141] or actively via demethylation by ten-eleven translocations (TETs) enzymes, which catalyse the oxidation of 5-methylcytosine to 5-hydroxymethylcytosine, 5-formylcytosine and 5-carboxylcytosine [142]. In an experimental model of mesothelioma development after rats exposure to asbestos, a significantly decreased expression of DNMT3a and 3b has been observed accompanied by increased levels of 5-hydroxymethylcytosine [143], indicating that epigenetic events are possible causes of activation of RNA editing downstream of activation of type 1 IFN.

## 6. Summary and open questions

In summary, ADAR-dependent RNA editing and ADAR expression, via their involvement in regulating type 1 IFN signalling, represent potential biomarkers and targets for mesothelioma immunotherapy for the treatment of mesothelioma, as has been shown for other cancers.

In addition, by their participation to RNA processing either through editing or by competing with other dsRNA-binding proteins, ADARs are involved in what has been called the 11th hallmark of cancer [144,145]. Although two studies have shown either the existence of ADAR editing in mesothelioma development [4] or growth suppression by silencing *ADAR2* in one mesothelioma cell line [53], most functional studies remain to be performed on this cancer type.

## Data accessibility

This article has no additional data.
